# Data on Income inequality in Germany, France, Italy, Spain, the UK, and other affluent nations, 2012

**DOI:** 10.1016/j.dib.2015.09.023

**Published:** 2015-10-09

**Authors:** Danny Dorling

**Affiliations:** School of Geography and the Environment, University of Oxford, UK

**Keywords:** Geography, Inequality, France, Germany, UK, Italy, Spain

## Abstract

This data article contains information on the distribution of household incomes in the five most populous European countries as surveyed in 2012, with data released in 2014 and published here aggregated and so further anonymized in 2015. The underlying source data is the already anonymized *EU Statistics on Income and Living Conditions (EUSILC) Microdata*. The data include the annual household income required in each country to fall within the best-off 1% in that country, median and mean incomes, average (mean) incomes of the best off 1%, 0.1% and estimates for the 0.01%, 0.001% and so on for the UK, and of the 90% and worse-off 10%, the best-off 10% and best-off 1% of households for all countries.

Average income from the state is also calculated by these income categories and the number of people working in finance and receiving over €1,000,000 a year in income is reported from other sources (the European Banking Authority). Finally income distribution data is provided from the USA and the rest of Europe in order to allow comparisons to be made. The data revealed the gross household (simple unweighted) median incomes in 2012 to have been (in order from best-off country by median to worse-off): France €39,000, Germany: €33,400, UK: €36,300, Italy €33,400 and Spain €27,000. However the medians, once households are weighted to reflect the nation populations do differ although they are in the same order: France €36,000, Germany: €33,400, UK: €31,300, Italy €31,000 and Spain €23,700. Thus weighting to increase representativeness of the medians reduces each by €3000, €0, €5000, €3300 and €3300 respectively. In short, the middle (weighted median) French household is €4700 a year better off than the middle UK family, and that is before housing costs are considered. This Data in Brief article accompanies Dorling, D. (2015) Income Inequality in the UK: Comparisons with five large Western European countries and the USA [1].

**Specifications table**

**Table:** 

Subject area	*Geography, Economics, Sociology, Politics, Health Sciences*
More specific subject area	*Income Inequality Measures*
Type of data	*Tables*
How data was acquired	*Data from anonymised EU Statistics on Income and Living Conditions (EUSILC) and the European Banking Authority*
Data format	*Analyzed*
Experimental factors	*Income distribution, comparison between EU countries.*
Experimental features	*Pretreatment of samples: data weighted by population size and for household charcteristics*
Data source location	*Europe, especially the five most populous countries of the EU: Germany, France, Italy, Spain, the UK, other affluent countries including Europe and the USA, Canada and Australia – summary statistics on the 1%*
Data accessibility	*Data is with this article*

**Value of the data**

• This data allows researchers to quickly examine household income distributions in large European countries without having to go through the formalities of accessing the raw data.
• Estimates are given for each of the five large countries of the number of households nationally living on incomes of many different thresholds so that it is possible to see the incomes of the best-off 1%,then the next best-off 1%, all the way down to the poorest 1%.
• European household incomes have been surveyed in a harmonized manner only relatively recently. Many researchers are unaware that this material is available, or that it reveals median incomes to be so much lower in the UK as compared to Germany and France.
• This data could inspire other works which might consider comparisons after housing costs have been taken into account or which might look in detail at more than five countries.

## 1 Data

Tables 1 and 2 give detailed breakdowns of gross household incomes in Euros in each of the five most populous countries of European countries. Please see the accompanying article [1] for associated tables.

## 2 Experimental design, materials and methods

The EU-SILC data was held in a spreadsheet containing 228,692 rows of information; one row for each household in Europe. Of these some 8058 were in the UK, 13,512 in Germany, 11,360 were in France, 19,399 were in Italy and 13,109 were in Spain. They were surveyed in 2012 and the data released to researchers in 2014. The following variables were used:

**Table:** 

1.	HY010	Gross household income
2.	HY020	Post-Tax household income
3.	HY022	Post-Benefits household income
4.	HY023	Post-Pension household income
5.	HY030G	Imputed rent gained from owning own property
6.	HY040G	Real rent gained from being a landlord to others
7.	HY050G	Child allowance income
8.	HY070G	Housing allowance income
9.	HY080G	Income received from alimony and similar sources
10.	HY090G	Profit – as income from owning shares and similar
11.	DB090	Weight – household weight used to sum households.

Simple excel functions such as this were used to calculate the various statistics shown in the tables

=SUMPRODUCT(C220635:C228692,$M220635:$M228692)/SUM($M220635:$M228692)

which sums the product of each pair of numbers C220635*M220635 down to the last pair and then divided that very large sum of the contained column M, which is the household weights. This is simply a weighted average.

To estimate top incomes a new method has to be introduced. First the geometric mean of three ratios was calculated as follows:

=GEOMEAN(C228704/C228705,C228703/C228704,C228702/C228703)

These are ratios of the worse-off 90% to the best-off 9% (less the top 1%); the 9% to the 1% (less the 0.1%); and the 1% to the 0.1% (less the 0.01% who are not in the surveys nor anyone better of than them). In the case of the UK this geometric mean was 3.2 meaning that each group tended to be 3.2 times more well-off than the group below it. Those ratios were assumed to remain constant resulting in a top annual income in the UK of £104million for the three richest households in 2012.

For Germany the ratio was 2.7 times and the very highest incomes estimated were €32.3 million. For France the ratio was 3.1 times and the very highest income was estimated to be €95.7 million (much less than the UK total which is in pounds and is nearer €125 million at the then exchange rates). For Italy the ratio was 2.7 times and the highest incomes were €34.8 million a year. For Spain the ratio was 1.9 times and the highest estimated annual incomes were €2.2 million.

Table 3 provides the figures used to calculate these ratios for all countries with the part of that table which became Table 2 in the paper this data brief accompanies highlighted in yellow. This paper needs to be read alongside the original research paper that first used the data described here to best understand why data in the form given here is needed.
