# Supplementary material for: Data on Income inequality in Germany, France, Italy, Spain, the UK, and other affluent nations, 2012
Source: Data Brief. 2015 Oct 9;5:458–60. doi: 10.1016/j.dib.2015.09.023 (PMC4610951; doi:10.1016/j.dib.2015.09.023)
Supplement: Supplementary file 2 — Supplementary material [file mmc2.zip › Table 3.docx]

**Table of data used to estimate very high incomes and summary statistics by groups – all 5 countries**

| Table 3: Summary of UK household income distribution 2012. | | | | | | | | |  |  |  |  |  |  |  |
| --- | --- | --- | --- | --- | --- | --- | --- | --- | --- | --- | --- | --- | --- | --- | --- |
| **UK** |  | Gross | Post-Tax | Post-Benefit | Post-Pen | Imp rent | Real rent | Child allowance | Housing allow-ance | Ali-mony etc | Profit | Weight |  |  |  |
| HB020 | HB030 | HY010 | HY020 | HY022 | HY023 | HY030G | HY040G | HY050G | HY070G | HY080G | HY090G | DB090 |  |  |  |
|  | Median | 30,267 | 25,458 | 22,469 | 13,481 | 4,180 | 0 | 0 | 0 | 0 | 0 | 2,602 |  |  |  |
| 8058 | Wmean | 43,909 | 33,121 | 29,809 | 23,808 | 3,281 | 527 | 1,032 | 715 | 179 | 553 | 3,270 | 2634808.513 |  |  |
|  | 1/10million | 104,386,279 | 56,138,025 | 56,138,025 | 56,138,025 |  |  |  |  |  |  |  | 0.00001% | 3 |  |
|  | 1/million | 32,988,872 | 17,741,126 | 17,741,126 | 17,741,126 |  |  |  |  |  |  |  | 0.0001% | 25 |  |
|  | 1/100,000 | 10,425,371 | 5,606,673 | 5,606,673 | 5,606,673 |  |  |  |  |  |  |  | 0.001% | 252 |  |
|  | 1/10,000 | 3,294,698 | 1,771,859 | 1,771,859 | 1,771,859 |  |  |  |  |  |  |  | 0.01% | 2517 |  |
| 8 | 1/1000 | 1,041,213 | 559,955 | 557,909 | 554,839 | 8,977 | 970 | 897 | 0 | 0 | 599 | 3,413 | 0.10% | 28,977 | 0.03% |
| 63 | 1/100 | 305,395 | 180,487 | 179,204 | 168,586 | 4,442 | 3,222 | 954 | 9 | 1,674 | 7,909 | 3,743 | 0.89% | 250,263 | 0.03% |
| 678 | WninePC | 116,942 | 80,326 | 78,828 | 74,533 | 2,325 | 2,092 | 905 | 28 | 85 | 1,658 | 3,495 | 8.99% | 2,514,896 | 0.03% |
| 7309 | WnintyPC | 32,863 | 26,332 | 22,817 | 16,688 | 3,359 | 343 | 1,045 | 792 | 174 | 370 | 3,245 | 90.01% | 25,169,665 | 0.03% |
| 782 | WtenPC | 7,671 | 6,835 | 4,358 | 194 | 3,920 | 53 | 212 | 993 | 128 | 104 | 3,367 |  |  |  |
| 8058 | Geomean | 3.2 | 2.8 | 2.9 | 3.2 |  |  |  |  |  |  |  | 100.00% | 27,963,800 | 0.03% |
|  | OnePercent | 420,648 | 240,639 | 239,290 | 229,553 |  |  |  |  |  |  |  |  | 97 | bankers/mil |
|  | 1%:median | 14 | 9 | 11 | 17 |  |  |  |  |  |  |  |  |  |  |
|  | 0.1%:10% | 136 | 82 | 128 | 2857 |  |  |  |  |  |  |  |  |  |  |
|  |  | 188,927 |  |  |  |  |  |  |  |  |  |  |  |  |  |
| **GERM-ANY** |  | Gross | Post-Tax | Post-Ben | Post-Pen | Imp rent | Real rent | Child allowance | Housing allow-ance | Ali-mony etc | Profit | Weight |  |  |  |
| HB020 | HB030 | HY010 | HY020 | HY022 | HY023 | HY030G | HY040G | HY050G | HY070G | HY080G | HY090G | DB090 |  |  |  |
|  | Median | 36,425 | 28,300 | 26,334 | 15,861 |  | 0 | 0 | 0 | 0 | 250 | 2,896 |  |  |  |
| 13512 | Wmean | 41,785 | 31,088 | 27,995 | 20,051 |  | 604 | 1,138 | 336 | 317 | 819 | 2,952 | 39889.97118 |  |  |
|  | 1/10million | 32,292,817 | 23,317,146 | 23,317,146 |  |  |  |  |  |  |  |  | 0.00001% | 4 |  |
|  | 1/million | 12,098,957 | 8,736,096 | 8,736,096 |  |  |  |  |  |  |  |  | 0.0001% | 36 |  |
|  | 1/100,000 | 4,533,044 | 3,273,101 | 3,273,101 |  |  |  |  |  |  |  |  | 0.001% | 361 |  |
|  | 1/10,000 | 1,698,369 | 1,226,313 | 1,226,313 |  |  |  |  |  |  |  |  | 0.01% | 3610 |  |
| 15 | 1/1000 | 636,318 | 459,455 | 413,080 | 366,473 |  | 68,215 | 1,815 | 0 | 101 | 57,444 | 2,654 | 0.10% | 40,032 | 0.04% |
| 137 | 1/100 | 194,957 | 131,540 | 126,054 | 117,367 |  | 7,270 | 2,253 | 0 | 271 | 5,170 | 2,628 | 0.90% | 362,023 | 0.04% |
| 1267 | WninePC | 103,060 | 69,554 | 66,379 | 61,568 |  | 1,458 | 2,053 | 5 | 170 | 1,757 | 2,832 | 8.99% | 3,608,308 | 0.04% |
| 12093 | WnintyPC | 33,466 | 25,762 | 22,748 | 14,542 |  | 377 | 1,035 | 373 | 332 | 618 | 2,969 | 90.00% | 36,104,738 | 0.03% |
| 904 | WtenPC | 7,656 | 6,874 | 2,588 | 839 |  | 38 | 190 | 1,441 | 611 | 116 | 4,412 | 10.00% |  |  |
| 13512 | Geomean | 2.7 | 2.6 | 2.6 | 2.9 |  |  |  |  |  |  |  | 100.00% | 40,115,100 | 0.03% |
|  | OnePercent | 257,067 | 177,388 | 167,925 |  |  |  |  |  |  |  |  |  | 5.28 | bankers/mil |
|  | 1%:median | 7 | 6 | 6 |  |  |  |  |  |  |  |  |  |  |  |
|  | 0.1%:10% | 83 | 67 | 160 |  |  |  |  |  |  |  |  |  |  |  |
|  |  | 153550 |  |  |  |  |  |  |  |  |  |  |  |  |  |
|  |  |  |  |  |  |  |  |  |  |  |  |  |  |  |  |
|  |  |  |  |  |  |  |  |  |  |  |  |  |  |  |  |
|  |  |  |  |  |  |  |  |  |  |  |  |  |  |  |  |
| **FRANCE** |  | Gross | Post-Tax | Post-Ben | Post-Pen | Imp rent | Real rent | Child allowance | Housing allow-ance | Ali-mony etc | Profit | Weight |  |  |  |
| HB020 | HB030 | HY010 | HY020 | HY022 | HY023 | HY030G | HY040G | HY050G | HY070G | HY080G | HY090G | DB090 |  |  |  |
|  | Median | 38,999 | 32,485 | 29,540 | 19,430 | 3,555 | 0 | 0 | 0 | 0 | 550 | 2,085 |  |  |  |
| 11360 | Wmean | 45,514 | 37,002 | 33,773 | 24,549 | 3,954 | 1,231 | 938 | 525 | 299 | 4,077 | 2,418 | 27470.74408 |  |  |
|  | 1/10million | 95,768,306 | 67,372,178 | 67,372,178 |  |  |  |  |  |  |  |  | 0.00001% | 3 |  |
|  | 1/million | 31,014,207 | 21,818,227 | 21,818,227 |  |  |  |  |  |  |  |  | 0.0001% | 25 |  |
|  | 1/100,000 | 10,043,834 | 7,065,751 | 7,065,751 |  |  |  |  |  |  |  |  | 0.001% | 250 |  |
|  | 1/10,000 | 3,252,658 | 2,288,217 | 2,288,217 |  |  |  |  |  |  |  |  | 0.01% | 2498 |  |
| 14 | 1/1000 | 1,053,361 | 741,030 | 737,196 | 676,653 | 13,097 | 42,706 | 1,340 | 325 | 404 | 469,009 | 2,018 | 0.10% | 28,540 | 0.05% |
| 111 | 1/100 | 254,822 | 187,687 | 185,149 | 173,366 | 9,462 | 16,641 | 1,320 | 66 | 127 | 49,339 | 2,224 | 0.90% | 249,417 | 0.04% |
| 1181 | WninePC | 110,521 | 85,598 | 82,070 | 69,078 | 7,055 | 4,788 | 1,077 | 90 | 319 | 14,597 | 2,092 | 8.99% | 2,496,010 | 0.05% |
| 10054 | WnintyPC | 35,776 | 29,837 | 26,631 | 17,868 | 3,579 | 674 | 920 | 573 | 299 | 2,043 | 2,459 | 90.00% | 24,976,432 | 0.04% |
| 918 | WtenPC | 10,695 | 10,005 | 6,670 | 2,142 | 2,050 | 85 | 173 | 1,144 | 330 | 422 | 2,993 | 10.00% |  |  |
| 11360 | Geomean | 3.1 | 2.9 | 3.0 | 3.4 |  |  |  |  |  |  |  | 100.00% | 27,750,400 | 0.04% |
|  | OnePercent | 375,063 | 271,336 | 268,692 |  |  |  |  |  |  |  |  |  | 6.38 | bankers/mil |
|  | 1%:median | 10 | 8 | 9 |  |  |  |  |  |  |  |  |  |  |  |
|  | 0.1%:10% | 98 | 74 | 111 |  |  |  |  |  |  |  |  |  |  |  |
|  |  | 188586 |  |  |  |  |  |  |  |  |  |  |  |  |  |
|  |  |  |  |  |  |  |  |  |  |  |  |  |  |  |  |
| **ITALY** |  | Gross | Post-Tax | Post-Ben | Post-Pen | Imp rent | Real rent | Child allowance | Housing allow-ance | Ali-mony etc | Profit | Weight |  |  |  |
| HB020 | HB030 | HY010 | HY020 | HY022 | HY023 | HY030G | HY040G | HY050G | HY070G | HY080G | HY090G | DB090 |  |  |  |
|  | Median | 33,389 | 26,332 | 24,777 | 16,795 | 6,102 | 0 | 0 | 0 | 0 | 0 | 1,035 |  |  |  |
| 19399 | Wmean | 39,862 | 29,817 | 28,250 | 20,057 | 5,610 | 1,060 | 261 | 30 | 135 | 316 | 1,300 | 25217.46208 |  |  |
|  | 1/10million | 34,789,397 | 21,285,186 | 21,285,186 |  |  |  |  |  |  |  |  | 0.00001% | 3 |  |
|  | 1/million | 12,777,360 | 7,817,568 | 7,817,568 |  |  |  |  |  |  |  |  | 0.0001% | 23 |  |
|  | 1/100,000 | 4,692,836 | 2,871,216 | 2,871,216 |  |  |  |  |  |  |  |  | 0.001% | 228 |  |
|  | 1/10,000 | 1,723,572 | 1,054,533 | 1,054,533 |  |  |  |  |  |  |  |  | 0.01% | 2280 |  |
| 28 | 1/1000 | 633,029 | 387,306 | 386,741 | 370,639 | 11,661 | 13,207 | 2 | 0 | 195 | 2,215 | 896 | 0.10% | 25,197 | 0.11% |
| 240 | 1/100 | 209,400 | 133,515 | 128,941 | 108,037 | 9,224 | 10,510 | 193 | 3 | 5 | 1,699 | 946 | 0.90% | 228,014 | 0.11% |
| 2033 | WninePC | 101,336 | 69,018 | 66,717 | 55,154 | 7,866 | 3,682 | 201 | 22 | 110 | 989 | 1,117 | 9.00% | 2,280,715 | 0.09% |
| 17098 | WnintyPC | 31,362 | 24,464 | 23,000 | 15,279 | 5,342 | 689 | 268 | 31 | 139 | 233 | 1,327 | 90.00% | 22,802,074 | 0.07% |
| 1719 | WtenPC | 6,280 | 5,672 | 5,041 | 1,477 | 4,304 | 109 | 66 | 46 | 141 | 78 | 1,468 | 10.01% |  |  |
| 19399 | Geomean | 2.7 | 2.5 | 2.6 | 2.9 |  |  |  |  |  |  |  | 100.00% | 25,336,000 | 0.08% |
|  | OnePercent | 270,099 | 170,067 | 165,933 |  |  |  |  |  |  |  |  |  | 4.30 | bankers/mil |
|  | 1%:median | 8 | 6 | 7 |  |  |  |  |  |  |  |  |  |  |  |
|  | 0.1%:10% | 101 | 68 | 77 |  |  |  |  |  |  |  |  |  |  |  |
|  |  | 164354 |  |  |  |  |  |  |  |  |  |  |  |  |  |
|  |  |  |  |  |  |  |  |  |  |  |  |  |  |  |  |
| **SPAIN** |  | Gross | Post-Tax | Post-Ben | Post-Pen | Imp rent | Real rent | Child allowance | Housing allow-ance | Ali-mony etc | Profit | Weight |  |  |  |
| HB020 | HB030 | HY010 | HY020 | HY022 | HY023 | HY030G | HY040G | HY050G | HY070G | HY080G | HY090G | DB090 |  |  |  |
|  | Median | 22,700 | 20,400 | 18,300 | 12,120 | 5,213 | 0 | 0 | 0 | 0 | 0 | 1,108 |  |  |  |
| 13109 | Wmean | 29,089 | 25,094 | 22,995 | 17,860 | 5,082 | 344 | 132 | 38 | 85 | 207 | 1,318 | 17279.16611 |  |  |
|  | 1/10million | 2,200,590 | 1,761,766 | 1,761,766 |  |  |  |  |  |  |  |  | 0.00001% | 2 |  |
|  | 1/million | 1,150,942 | 921,430 | 921,430 |  |  |  |  |  |  |  |  | 0.0001% | 16 |  |
|  | 1/100,000 | 601,960 | 481,922 | 481,922 |  |  |  |  |  |  |  |  | 0.001% | 157 |  |
|  | 1/10,000 | 314,834 | 252,052 | 252,052 |  |  |  |  |  |  |  |  | 0.01% | 1569 |  |
| 17 | 1/1000 | 164,663 | 131,827 | 131,449 | 126,076 | 7,988 | 10,715 | 0 | 0 | 0 | 3,351 | 1,043 | 0.10% | 17,890 | 0.10% |
| 101 | 1/100 | 119,420 | 95,589 | 92,749 | 84,394 | 7,292 | 1,709 | 171 | 0 | 27 | 3,153 | 1,539 | 0.90% | 156,847 | 0.06% |
| 1171 | WninePC | 73,826 | 60,731 | 58,361 | 53,128 | 6,417 | 1,181 | 398 | 33 | 104 | 715 | 1,328 | 9.00% | 1,569,001 | 0.07% |
| 11820 | WnintyPC | 23,558 | 20,703 | 18,637 | 13,545 | 4,923 | 235 | 105 | 39 | 83 | 123 | 1,316 | 90.00% | 15,689,962 | 0.08% |
| 1381 | WtenPC | 3,916 | 3,592 | 2,246 | 65 | 4,336 | 52 | 41 | 19 | 96 | 41 | 1,251 | 10.00% |  |  |
| 13109 | Geomean | 1.9 | 1.9 | 1.9 | 2.1 |  |  |  |  |  |  |  | 100.00% | 17,433,700 | 0.08% |
|  | OnePercent | 126,285 | 101,087 | 98,525 |  |  |  |  |  |  |  |  |  | 5.74 | bankers/mil |
|  | 1%:median | 6 | 5 | 5 |  |  |  |  |  |  |  |  |  |  |  |
|  | 0.1%:10% | 42 | 37 | 59 |  |  |  |  |  |  |  |  |  |  |  |
|  |  | 105249.35 |  |  |  |  |  |  |  |  |  |  |  |  |  |
